# Diabetes, Care Homes, and the Influence of Technology on Practice and Care Delivery in Care Homes: Systematic Review and Qualitative Synthesis

**DOI:** 10.2196/11526

**Published:** 2019-04-22

**Authors:** Rebecca Mathews, Chris O'Malley, Jenny M Hall, Leah Macaden, Sandra MacRury

**Affiliations:** 1 Division of Rural Health and Wellbeing Centre for Health Science University of the Highlands and Islands Inverness United Kingdom; 2 Highland Health Sciences Library Centre for Health Science University of the Highlands and Islands Inverness United Kingdom; 3 Department of Nursing Centre for Health Science University of the Highlands and Islands Inverness United Kingdom

**Keywords:** diabetes mellitus, technology, residential facilities, nursing homes

## Abstract

**Background:**

Diabetes is increasing in prevalence and complexity in the care home setting, affecting up to a quarter of care home residents. Health outcomes for these residents are impacted by management of the disease, health care professionals (HCPs)’ decision-making skills within the care home setting, and access to specialist services. The use of technology has the potential to recognize opportunities for early intervention that enables efficient responsive care, taking a fundamental role in linking the care home community to wider multidisciplinary teams for support.

**Objective:**

The aim of this paper was to identify evidence that explores factors relevant to the use of technology in and around the care home setting to aid in the management of diabetes.

**Methods:**

Databases searched using a structured prespecified approach included: PubMed, CINAHL (Cumulative Index to Nursing and Allied Health Literature), OVID Nursing database, Scopus, MEDLINE, the Cochrane Library, and the King’s Fund from 2012 to 2017: handsearching was undertaken additionally for any gray literature. Preferred Reporting Items for Systematic review and Meta-Analysis Protocol was used as protocol with Risk of Bias in Systematic reviews a tool to assess the risk of bias across studies. Studies had to include interventions that combined technology to or from the care home setting to support residents living with diabetes.

**Results:**

The combined search strategy identified a total of 493 electronic records. Of these, 171 papers were screened for eligibility, 66 full papers were accessed, and 13 have been included in this study. Qualitative synthesis has identified different strands of research evidence in what and how technology is currently being used in and around care homes to enhance diabetes management. New initiatives and implementations of technology and emerging models of care that included the use of technology have also been included.

**Conclusions:**

By triangulating the perspectives of HCPs, practitioners, specialists, and members of the care home community, the authors anticipate that this review will represent an up-to-date, evidence-based overview of the potential for using technology within the care home setting for diabetes management as well as stimulate research in this area.

## Introduction

### Background

The prevalence of diabetes is increasing in parallel with population aging within the Scottish Highlands [1-3]. It has been identified that more than a quarter of care home residents have some kind of diabetes, whether diagnosed or undiagnosed [4-6] bringing challenges for nursing practice and specialist care services, accordingly, the need to access specialist services is increasing. The population in Scotland is projected to rise by 7% by 2039, with an increase of 85% in those living over the age of 75 years [7]. With huge increases predicted in the prevalence of diabetes in elderly persons, and the likelihood of developing diabetes as they age [8], management of this disease in the care home setting is of paramount importance.

Access to specialist services for care home residents living with comorbidities is limited [9] leaving care home staff continually seeking to deliver treatments more independently, sometimes with expectations and demands more than they feel competent to provide. Residents may be dependent on care home staff in relation to interventions around diabetes management, particularly with tasks such as blood glucose monitoring or insulin injections [10]. Most nursing home residents are unable to initiate access to doctors or community health care independently or make their own decisions about self-care [1,4,11]; therefore, care home staff must become both advocates and facilitators of care interventions. Today, the advancement of technology has the potential to influence practice in the delivery of safe, effective, quality, and seamless care [12] as well as to promote communication and provide access to multidisciplinary specialist care [13] aiding clinical support for residents within the care home community. Moreover, there is a need for trained staff to lead care planning, initiate treatment, and make independent decisions to support those living with diabetes. In turn, this should empower care home staff to contribute to better diabetes management, thus raising the standards of care within the care home setting. Residents living with diabetes represent one of the most difficult challenges to health professionals and care home staff in advancing care [14] and may need to receive care from multiple medical services. Given that the complications of diabetes and the associated comorbidities alongside the aging process [1] make residents potentially more vulnerable, care home staff are challenged in the provision of care to manage the disease effectively and influence outcomes.

To support staff and residents in care homes, clear structure and collaboration between care home and health care services is of importance. Standardized education and training in relation to diabetes care and management for care home staff would aid this process, thus reducing complications and improving quality of care for residents [15]. This should include clear access with good communication and support between specialists, primary and secondary services, and the implementation of defined standards of care for residents living with diabetes. Diabetes UK [16] has recognized the importance of providing standards of care for residents in care homes and published guidance in 2010 setting standards for diabetes care in residential homes, and multiple care home–specific policies, statements, guidelines, and recommendations [5,6,17-20] exist to support this approach. Nevertheless, at least in the Highlands in Scotland, there is currently no mandatory diabetes training for staff in care homes, no defined standards of care for residents living with diabetes, and no clear protocol for accessing specialist services in the care home setting, highlighting the need for tighter regulation to improve care for those living with diabetes in care homes [14].

There are many emerging and existing solutions to the provision of better support for health care professionals (HCPs) incorporating the use of technology within care delivery. The Joint Asia Diabetes Evaluation program advocates a nurse doctor team with a Web-based portal that uses care protocols and a validated risk engine to provide decision support and regular feedback [21]. Taking technology-enabled care (TEC) services forward, lessons need to be learned from good practice to adopt long-term organizational change on care pathways for the management of long-term conditions [22]. Health information technology has been shown to assist in decision support, improving care co-ordination, communication and therefore outcomes for older adults [23,24]. Electronic-health technologies, electronic health records, electronic medical records, and the use of electronic decision support enabling collaboration of care, communication and information sharing between HCPs [25,26] have been found to have a positive impact in caring for people living with diabetes [27] by enabling an overview of clinical information and prompts for diabetes care and management. The Informatics for Diabetes Education and Telemedicine project individualizes care by using video conferencing (VC), demonstrating a centralized support approach in the use of technology to promote education with personalized behavior goal setting accomplished through televisits with a nurse manager and dietitian [28]. Despite poor technology infrastructure and lack of user-friendly technology training reported by Kolltveit [29], it is also recognized that the use of telehealth technology holds considerable potential in the care home setting, enabling both proactive and reactive approaches, teamwork, partnership, and harmonization between allied HCPs granting distant interaction, working in different settings promoting communication, and, therefore, enabling early implementation of interventions [29,30]. Furthermore, the importance of the use of technology to aid social interaction for older people remains, with evidence suggesting that older people can successfully learn new technological skills, enhancing the quality of life while being mentally alert and engaged with wider communities [31-33].

### Objective

This study aimed to systematically review the literature to identify evidence relevant to the use of technology in and around the care home setting to aid in the management of diabetes as well as to explore the nature of technologies and how they are being used to support staff and residents living with diabetes, identifying and synthesizing existing and new models of care that hold potential to enhance care and aid management in care homes.

## Methods

For the purpose of this study, and to clarify, the words *care home community* will relate to individuals associated with a care home, nursing home or residential home. These may include residents, carers, managers, staff, nurses, and those who are connected to the home.

### Eligibility Criteria

Studies were selected according to the criteria outlined below.

### Study Design

Study characteristics of published or unpublished, controlled or uncontrolled research study design including both qualitative and quantitative studies; reports; and case studies have been selected. Additional methods to capture further studies included handsearching and reference and citation checking, which were undertaken by author 1 (RM).

#### Included Studies

We considered interventions using any type of technologies provided by, to, or from the care home setting, that had an influence on practice assisting in care delivery and interventions that involved engagement with specialist services, ongoing treatment management, or communication with wider health services. Also of interest were new and emerging technological models of care that provided support for long-term condition management currently being used in different health regimens that could be applied to the care home setting. Studies included were dated from 2012 to 2017. Articles were not restricted to English language. One article of another language was translated by Microsoft Edge, developed by Microsoft.

#### Excluded Studies

We excluded studies that involved any form of self-management technology, self-monitoring and self-reporting, mobile digital platforms, community setups, care at home, home care services, telecare assistive devices, mobile phone technology, smart care, smartphones, and smart homecare technology.

### Participants

Care home managers, staff, residents, patients, carers, older persons, and those representing the care home community as well as General Practitioners, specialists, experts, nurse practitioners, allied HCPs, and those with a specific interest in diabetes management were included in this study.

### Types of Interventions

We considered any interventions using any types of technologies provided to, or from the care home setting; the influence of technology on clinical practice and care delivery to or from the care home; and interventions that involved engagement with specialist services, ongoing treatment, management, or communication with wider health services.

### Information Sources

To ensure literature saturation, 7 electronic bibliographic databases holding peer-reviewed publications of specialist research design and trusted evidence in health care were identified for their relevance. The databases included were MEDLINE, CINAHL (Cumulative Index to Nursing and Allied Health Literature), the Cochrane Library, PubMed, the King’s Fund, OVID nursing base, and Scopus.

### Search Strategy

To illustrate methodological rigor in this systematic review, a robust protocol, the Preferred Reporting Items for Systematic Review and Meta-Analysis (PRISMA P 2015) [34] (Multimedia Appendix 1) was used for all potentially relevant articles to enhance quality, transparency, and strength. This approach provides an explicitly planned document supporting consistent evidenced-based research integrity in health care as well as reduced duplication of effort. In addition, Risk of Bias in Systematic reviews (ROBIS) 2016 [35], a new tool designed to specifically assess the risk of bias in systematic reviews has been utilized.

#### Electronic Search Strategy

This specific search strategy has been developed, performed, reviewed, and completed by author 1 (RM) with input from a Highland Health Sciences Librarian (CoM), not otherwise associated with the project, but with expertise in systematic review searching to ensure legitimacy. The structured databases were searched using a combination of subject headings and keywords. Concepts were banded together as described in Table 1, with the intent of covering the range of inclusion criteria for searching, to provide consistency in the searches across the databases. A list of the databases is provided in Table 2.

The King’s Fund was contacted directly after performing a basic search using the keywords: diabetes, technology, care homes and telehealth. This specific database developed an advanced search using keywords that were then adapted into broader categories by the King’s Fund: (1) care homes and technology; (2) diabetes and technology in health and social care; and (3) telehealth, telecare, and telemedicine. Textbox 1 shows an example search for one database.

**Table 1:** Electronic search terms and clustering of components.

Diabetes OR Diabetes Mellitus	Diabetes type 1 OR Diabetes type 2	DKA OR Hetoacidosis	Hypo OR	Hypoglycemia
AND Technology OR	AND Residential care OR Nursing care OR	AND Specialist Services OR	AND Residential home Nursing home Care home OR	AND Nursing home community OR
Email	Delivery of care	Integrated	Access to services	Care home staff
Laptop	Specialist care	Interdisciplinary	Support	Nurse
Tablet	Specialist nurse	Integra	Guidelines	Senior nurse
Video conference	Continuity of care	Seamless	Diabetes knowledge	Senior nurse carer
Digital	Primary care	Interprofessional	Educational package	Senior nurse care assistant
eHealth	Secondary care	Shared	Resources	Carer
Telephone	Service delivery	Model of care	Frameworks	Nursing home staff
Personal computer	Manage	Community	Protocols	Care assistant
Educational program	Management	Information sharing	Policy or policies	Support worker
Telehealth	Intervention	Collaborative	Information	

**Table 2:** Database table.

Database	Date search complete	Papers identified and titles screened	Duplicate papers	Result and total number of abstracts screened	Full papers accessed	Studies included (N)
PubMed via the National Center for Biotechnology Information	April 3, 2017	60	10	37	1	1
CINAHL via EBSCO	April 3, 2017	23	2	5	5	1
Scopus via Elsevier	April 3, 2017	118	8	35	11	3
OVID nursing	April 3, 2017	3	0	0	0	0
MEDLINE via EBSCO	April 3, 2017	1	1	1	1	0
The Cochrane Library	April 3, 2017	Basic search: 9	3	3	0	0
	April 3, 2017	Advanced search: 99	6	22	8	1
The King’s Fund	April 3, 2017	Basic search: 3	1	3	1	3
	June 9, 2017	Advanced search: 50; 55; 52	3	12; 10; 23	5; 12	3
Additional searches	April to August 2017	20	0	20	8	2; 1 (to be published)
Total	—^a^	493	34	171	66	13

^a^Not applicable.

**Database search for Scopus.**

(TITLE-ABS-KEY (diabetes OR “diabetes mellitus” OR “diabetes type 1” OR “diabetes type 2” OR “diabetes type one” OR “diabetes type 2” OR dka OR hypoglycaemi* OR ketoacidosis)) AND ((TITLE-ABS-KEY((technolog* OR email OR laptop OR tablet OR telephone OR digital OR ehealth OR videoconference OR vc OR pc OR “ personal computer” OR telehealth OR “educational program””))) AND (TITLE-ABS-KEY((“resident”* care” OR “nurs* care” OR “delivery of care” OR “specialist care” OR “specialist nurse” OR “continuity of care” OR “primary care” OR “secondary care” OR “service delivery” OR “manag” OR interven*))) AND (TITLE-ABS-KEY ((“specialist care” OR integrated OR interdisciplin* OR intergrat* OR seamless OR inter*profession* OR shared OR “model of care” OR community OR “information sharing” OR collaborative))) AND (TITLE-ABS-KEY((“resident* home” OR “nursing home” OR “care home” OR “access to services” OR support OR guideline* OR framework* OR protocol* OR resource* OR “diabetes knowledge” OR “education* package” OR policy OR policies OR information))) AND (TITLE-ABS-KEY((“nursing home community” OR “care home staff” OR nurse OR “senior nurse” OR “senior nurse carer” OR snca OR carer OR “nursing home staff” OR “care assistant” OR “support worker”)))) AND (LIMIT TO (PUBYEAR2017) OR LIMIT- TO (PUBYEAR , 2016) OR LIMIT-TO (PUBYEAR, 2015) OR LIMIT-TO PUBYEAR ,2014) OR LIMIT-TO (PUBYEAR, 2013) OR LIMIT-TO PUBYEAR, 2012))

### Study Records

#### Selection Process

**Figure 1:**
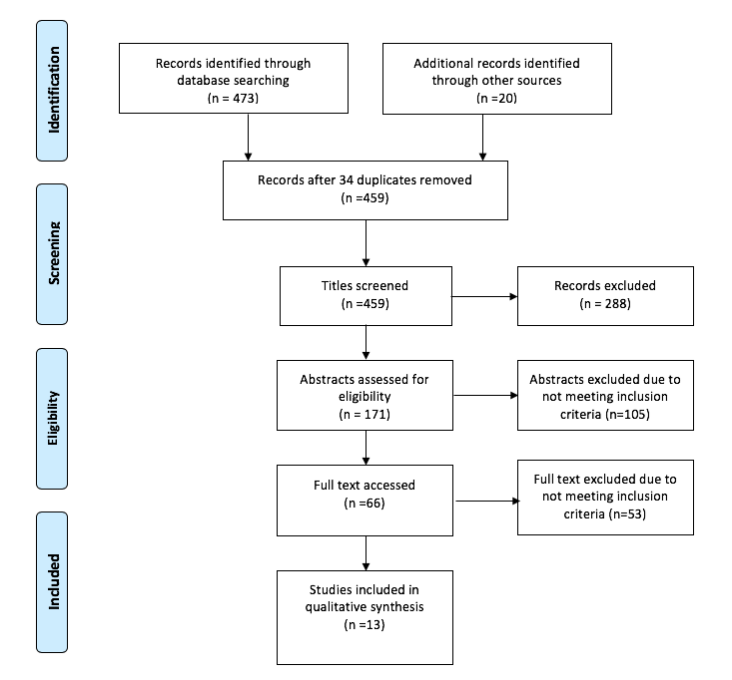
Selection process using Preferred Reporting Items for Systematic review and Meta-Analysis (PRISMA P 2009).

To enhance objectivity, 2 reviewers (authors 1 and 3) independently screened titles of electronic records generated by the search against the inclusion and exclusion criteria. Full abstracts were obtained for all the titles that appeared to meet the inclusion criteria. This process is shown in Figure 1 [36]. Author 1 independently reviewed abstracts followed by an independent review by author 3. Authors 1 and 3 were not blind to the journal titles or study authors. For neutral papers, decisions were made through discussion, and full-text articles were then accessed for inclusion.

#### Data Collection Process

To extract relevant data from included studies, a data extraction form was developed before the search by author 1, adapted and based on an extended PICO (population, intervention, comparison, outcome) design [37-40] as a framework before proceeding with qualitative synthesis to identify themes (Multimedia Appendix 2). Elements of the studies addressed the following: author, study year, country, title, group covered/participants, study design, aim or discussion of the project or service, intervention, key findings, and planned or actual effectiveness. Individual studies or reports that consisted of multiple interventions were combined.

### Outcomes and Prioritization

#### Primary Outcomes

The primary outcomes were to pull together existing forms of technology that are being designed to facilitate integrated working between allied health professionals and the care home community, and to highlight and expand the understanding of the use of technology-enabled approaches to diabetes management within the care home setting.

#### Secondary Outcomes

The secondary outcomes included informing health care policy decisions by standardizing diabetes education and training within the care home setting for staff and bringing in defined standards of care for those residents living with diabetes. Undertaking evidence reviews for new and emerging models of care will help us to understand preconditions for success, learning from other care settings and countries.

### Risk of Bias of Individual Studies

To determine the risk of bias within included studies and identify any concerns with the review process, the methodological quality of studies was assessed using ROBIS 2016. As bias can arise at all stages of the review process, assessment was executed for each study throughout using the ROBIS tool and was completed in 4 domains as shown in Table 3. However, no studies have been excluded based on the quality of bias. Phase 1: assessing relevance: this process was covered by devising a prespecified data extraction form adapted and based on an extended version of PICO. Phase 2 identified concerns with the review process and phase 3 judges the overall risk of bias and are shown in Table 3.

**Table 3:** Determining Risk of Bias of Studies

Domain	1. Study eligibility criteria	2. Identification and selection of studies	3. Data collection and study appraisal	4. Synthesis and findings	Risk of bias in the review
Signaling questions	1.1 Did the review adhere to predefined objectives and eligibility criteria?; 1.2 Were the eligibility criteria appropriate for the review question?; 1.3 Were eligibility criteria unambiguous?; 1.4 Were all restrictions in eligibility criteria based on study characteristics appropriate?; 1.5 Were any restrictions in eligibility criteria based on sources of information appropriate?	2.1 Did the search include an appropriate range of databases/electronic sources for published and unpublished reports?; 2.2 Were methods additional to database searching used to identify relevant reports?; 2.3 Were the terms and structure of the search strategy likely to retrieve as many eligible studies as possible?; 2.4 Were restrictions based on date, publication format, or language appropriate?; 2.5 were efforts made to minimize error in selection of studies?	3.1 were efforts made to minimize error in data collection?; 3.2 Were sufficient study characteristics available for both review authors and readers to interpret the results?; 3.3 Were all relevant study results collected for use in the synthesis?; 3.4 Was risk of bias (or methodologic quality) formally assessed using appropriate criteria?; 3.5 were efforts made to minimize error in risk of bias assessment?	4.1 Did the synthesis include all studies that it should?; 4.2 Were all relevant study results collected for use in the synthesis?; 4.3 Was the synthesis appropriate given the nature and similarity in the research questions, study designs, and outcomes across included studies?; 4.4 Was between-study variation minimal or addressed in the synthesis?; 4.5 Were the findings robust, for example, as demonstrated through funnel plot or sensitivity analysis?; 4.6 Were biases in primary studies minimal or addressed in the synthesis?	A. Did the interpretation of findings address all the concerns identified in domains 1 to 4?; B. Was the relevance of studies to the reviews research question appropriately considered?; C. Did the reviewers avoid emphasizing results on the basis of their statistical significance?
Judgement	Concerns regarding specification of study eligibility criteria	Concerns regarding methods used to identify and/or select studies	Concerns regarding methods used to collect data and appraise studies	Concerns regarding the synthesis	Risk of bias in the review

## Results

### Overview

A summary of all the information studies is provided in Table 4.

### Data Synthesis

#### Summary of Using Risk of Bias in Systematic Reviews

Due to the diversity of mixed-methods studies included in this analysis, the use of ROBIS 2016 proved challenging. The risk of bias was rated as low for 10 studies; the findings from these studies are *likely to be reliable* according to ROBIS. Phase 2 did not raise concerns with the review process; concerns were identified by the authors and addressed in the study conclusions. For the 3 studies viewed as the unclear risk of bias, these included reports of various implementations of telehealth, telemedicine, and telecare, such as TEC services across the United Kingdom, including case studies, pilot programs and vision statements; therefore, there was insufficient information reported to make a judgment on biases. However, these studies were included in the synthesis.

**Table 4:** Summary of information of studies.

Study; location	Groups, participants covered and sample size	Study design	Aim or discussion	Types of technology intervention	Key findings and planned or actual effectiveness
Study 1: Fox et al (2013) [41]; United Kingdom	Care home managers; Senior nurses from care homes; Health care assistants from care homes; Domiciliary managers from care homes; Combined sample of n=779 (20 studies)	Mixed-methods approach	Diabetes management in care homes and the use of guidelines for good standards of care in residential and nursing homes in the United Kingdom	Workshops led by health care professionals including focus group to identify key educational needs; educational presentation using VC^a^; and electronic learning	Identification of lack of written policy for diabetes management, knowledge, and training for staff in diabetes care, inadequate assessment of residents, lack of communication and specialist support from diabetes team, lack of resources for identification of risk of diabetes, access to specialist services, care planning and quality indicators. Educational needs supported by electronic learning seen as an inferior form of education when compared with a skilled, experienced educator.
Study 2: Benetos et al (2013) [42]; France	Elderly, frail residents in care homes living with diabetes and related comorbidities; sample size n=339 and n=675 (15 studies)	Mixed-methods approach, compilation of simple pragmatic advice for HCPs.	Initiatives for diabetes management in nursing homes.	Pragmatic advice for HCPs^b^ concerning the management of elderly, frail diabetic patients tailored by a multidisciplinary team of French experts; eye slit lamp technology used by visiting HCP to the care home	To prevent complications associated with diabetes and comorbidities with emphasis on personal individual care planning for residents to adapt and individualize treatment together with education and training of patient, family, physician, nurses, and carers including individualized monitoring of glycemic targets. Access to specialists is recommended to be onsite.
Study 3: Kilvert & Fox (2015) [43]; United Kingdom	Older people living with diabetes with reference to the care home setting; sample size audit: 49,000; 2 case studies	Case studies, focus groups, and audit.	Recommendations and acknowledgment of policies and guidelines relating to diabetes management.	Improved technology using smart glucose meters; basal bolus regimes; insulin pumps and CGM^c^.	CGM could be employed to alert carers of undesirably low or high blood glucose levels, recognition of the need to individualize treatment, inability to access education, and training and communication between care home and primary care poor. Guidance produced by Diabetes UK
Study 4: Hausken & Graue (2013) [44]; Norway	HCPs in nursing homes who care for older people with diabetes; sample size: 20 HCPs	Mixed-methods approach	Adequate training and support for enhanced professional competence of diabetes management in nursing homes and home-based services.	Educational training using a pilot program for health care workers; online learning and Web-based technology a potential source.	In Norway, it is a legal requirement to educate specialist health care workers to promote enhanced professional competence and ensure knowledge transfer between HCPs. Nurses and nursing aides are recognized as having different educational needs. This program provided enhanced evidence-based practice skills. Further research is recommended in this area.
Study 5: NHS England (2015) [45]; United Kingdom	Care homes and the care home community; sample NHS^d^ reports and case studies about care services in England representing 9 areas across the United Kingdom (Sunderland, Nottingham-shire, London, Sussex, Stoke-on-Trent, Shropshire, Telford, Calderdale, Airedale, and Blackpool)	Case studies and reports	Various implementations to develop technological initiatives between care home and care provider to ensure multidisciplinary team engagement and reshape how care is delivered across England	Teleswallowing to provide Remote Assessment Providing Interventions for Dysphagia (RAPID); telehealth system using a tablet to aid caregiver using the National Early Warning Score (NEWS) as monitoring tool across the board; Using FLO with Skype by live streaming with General Practitioners’ for ward rounds. Promoting communication by fax, android tablet, two-way video links, telecare monitoring equipment with online toolkits for all HCPs. Secure email accounts; shared directory created; standardized documentation with structured messages developed to a national care standard to ensure MDT^e^ engagement and support with each other. Android tablets for monitoring telehealth system alerting staff early to initiate early interventions aiding to avoid admission to hospital. Care homes using secure email to improve the flow of information and communication in and out of care homes; online toolkit for staff; telehealth system to alert changes in patients’ observations, for example, monitoring the risk of falls. Two-way video link between care home and clinicians.	Various implementations of telehealth, telemedicine, and telecare including technology-enabled care services across the United Kingdom between care homes and care providers. New and proactive ways of the delivery of safe, effective care.
Study 6: Carlisle & Warren (2013) [30]; Australia	Health practitioners and patients; sample size; 2-arm prospective RCT^f^; n=210	Mixed-methods	To explore the implementation of a telehealth service within a coordinated model of care for chronic disease management.	Telehealth using home monitoring and videoconferencing with the diabetes care coordinator and GP^g^ to resolve emerging clinical complications; regular emailed reports; broadband communication; telephone contact as required	Definitive conclusions not possible because of limited sample size. However, results showed that participants were keen to engage in telehealth, interpersonal skills, and operational factors were identified as key enablers. Positive working relationships were identified as important for sustaining engagement with telehealth. Benefits of telehealth such as greater access to health care services, improved health outcomes, and effective service delivery. It also highlighted the complexity of chronic disease management positively influenced by the effective implementation of telehealth.
Study 7: Cook et al (2017) [46]; United Kingdom	Residential and nursing home community; GPs and allied health care professionals; sample size (study 1) n=45; (study 2) n=28	Qualitative using constructionist methodology	Aligning access to GP, practice and older people, nurse specialists with care homes using the whole systems service delivery model approach.	Enhanced health care infrastructure for care home residents using a community-based virtual ward providing regular case management. MDT health professionals are then drawn into the group on a case-by-case basis.	Multiple competencies are required by the HCP to provide preventative care, the complex management of frailty, comorbidity, and end-of-life care can only be achieved through multisector and multi-professional working. The whole systems approach enables practitioners to share information and knowledge, problem solve and deliver coordinated care.
Study 8: Brown et al (2016) [47]; United States	NPs^h^ and RNs^i^ caring for the older population of patients with long-standing diabetes; sample size n=52	Quality improvement project and pilot intervention longitudinal cohort evaluation	New model of diabetes management in chronic care delivery.	Care coordination using telehealth, for education and virtual outreach clinic; Communication between veterans and health care providers using protocols and documentation template; registered nurses provided patient education coaching in addition to protocol-adjusted medications; and transmission of blood glucose management via telephone	RNs use of didactic training, access to NP team leader partnering with patients, the use of a mentor, the use of medication titration using protocols and support from a clinical team to ensure safe, timely, and efficient care.
Study 9: Spanakis et al (2012) [48]; Europe	Allied professionals, patients, and specialists; sample: doctors, nurses, social scientists, technical personnel, patients, carers, nutritionists, and lawyers	Qualitative using focus groups	New care models incorporating advanced ICT^j^ to support diabetes management in clinical applications CGM in different health care regimes, to integrate clinical and organizational workflows with external health information systems.	REACTION Platform via the Web to integrate care; insulin pumps; electronic health for integrated care space; closed-loop system aiding management of diabetes using ICT; glucose monitoring system; ICT tools for health care support; feedback provision to the point of care; integrative risk assessments Web-based; and electronic patch sensors	Technology allowing for a more accurate, faster response to crisis as well as better overall management in the prevention of complications of the disease. Advanced ICT and wireless technologies to enable continuous monitoring and automated closed-loop delivery of insulin via an insulin pump. The REACTION platform endeavors to provide integrated, professional, management, and therapy services using new chronic care models for diabetes patients in and across Europe.
Study 10: Wild et al (2014) [49]; United Kingdom	Care home community including staff, relatives, residents, and carers attached to the care home; sample care staff: n=20; residents n=10; and relatives n=10	Qualitative	Views and perceptions of the care home community on the role of technology design and the potential value of using technology for the care of older residents.	Future development of technologies within care homes considering: environment; assistive devices links to the community associated with a therapeutic approach; assessment tools; links to MDT; telecare; wireless sensors; telehealth; and virtual external access; speech recognition	Considerations for the development of design and technology, preparation for the introduction of technology would increase uptake, older people can learn new technological skills. To create technology that recognizes residents’ long-term care needs enabling and empowering residents with the aim of improving the overall quality of life. Lack of research in this area is a limitation.
Study 11: Mason (2012) [50]; United Kingdom	Care Home Community; sample workshop participants	Report: Vision of the Care Home of the Future	Central to meeting the needs of older people in care homes, with a focus on the delivery of care using technology to improve the lives of residents.	Technology for staff supporting care delivery in the care home setting. Technology to keep people mentally alert and engaged with the outside world; checking systems for staff and residents; tablets and computers; GPS^k^ trackers; medication reminders; epilepsy monitors; lifestyle and behavioral monitoring; video conferencing; telemedicine; information sharing via the Web; and environmental technologies technology tailored to meet individual needs	Future aspirations and visions for the future of care homes to primarily improve the quality of lives of residents promoting changing the landscape of care homes, workshops, staffing developments, care regulation and environment. A clear vision for the care home setting to be seen as a community hub enabling world-class coordinated leading quality care.
Study 12: Brittain et al (2016) [51]; United Kingdom	Care home community; 761 studies mapped	Rapid evidence synthesis	To underpin the spread of new models of care by conducting a rapid synthesis of evidence relating to enhancing health in care homes.	Technology: cost, ease of use, and staff demands; Workforce: interventions promoting positive joint working within the care home; Communication and engagement- tools to structure communication have the potential to enhance clinical outcomes; and Evaluation: insufficient data reported.	Digital technology has multiple potential applications in care homes. The use of SBAR^l^ as a standardized tool to structure communication. Cost, ease of use and staff demands identified as both barriers and facilitators to the implementation and use of technology.
Study 13: Benhamou et al (2013) [52]; France	Patients living with diabetes; sample: n=163, older diabetic individuals	Mixed-methods review	Current developments of information technology for the management of diabetes	Telemedicine delivery platform involving doctors and nurse educators; telemonitoring; Web-based programs to enable collaborative access to patient medical records. Downloadable capillary glycemic data; feedback with GPs via telephone consultations enabling real-time decision support; blood glucose online diary; secure messaging system; and educational websites	The use of information tools, specific software, and the internet provides support and encourages behavior necessary to prevent clinical inertia and effectively manage diabetes by improvement of therapeutic compliance through motivational support. The development of telemedicine and mobile internet contributes to better diabetes management for the user.

^a^VC: video conferencing.

^b^HCP: health care professional.

^c^CGM: continuous glucose monitoring

^d^NHS: National Health Service

^e^MDT: multidisciplinary team.

^f^RCT: randomized controlled trial.

^g^GP: general practitioner.

^h^NP: nurse practitioner.

^i^RN: registered nurse.

^j^ICT: Information and Communication Technologies.

^k^GPS: Global Positioning System.

^l^SBAR: Situation, Background, Assessment, Recommendation.

## Discussion

### Search Outcome

This systematic review synthesizes the database results of relevant studies in the use of technology in and around the care home setting to support the care home community and staff responsible for the care of residents living with diabetes. Considerations for the development of design and technology included views and perceptions of the care home community on the role of technology design and the potential value of using technology for the systematic management of diabetes in older residents. Studies highlighted that there is a comprehensive role for co-design in the way that technology can be used within the care home setting for empowering care homes. Supporting technologies and monitoring devices are being used together with telephone calls, interactive Web-based management systems, educational websites, and multidisciplinary communication. This review highlights that technology solutions are being sought and used; however, uptake of the use of technology is slow to progress and training and support in the use of telehealth technology is crucial to aid HCPs.

Most studies included multifaceted interventions necessary to effectively manage diabetes. However, despite the broad inclusion criteria, limited research was found in this area. It is clear that multiple competencies are required by care home staff to individually manage cases of elderly residents living with diabetes; fundamentally, it is knowledge, skills, and the ability of care home staff to access specialist support and services that affect the quality of care for residents helping to prevent clinical inertia in diabetes management including care coordination and feedback provision to the point of care and standardized tools to structure communication [48,51,52]. To prevent complications associated with diabetes and comorbidities, personal individual care planning for residents to adapt and individualize treatment initiatives for diabetes management in nursing homes was emphasized [42].

Educational needs for care home staff are being recognized and supported [41-44,47] by using technological interventions such as VC alongside educational workshops, pilot programs, electronic learning (e-learning), and websites [41,45,48,49,52] including Web-based learning and information communication toolkits [44,45]. The significance of design and support is recognized within studies rating the suitability of blended learning as an approach to education, including a combination of hands-on skills-based training from experts to enhance evidence-based practice skills in addition to using Web-based or e-learning facilities.

Technology being used in the care home setting by visiting HCPs was also reported alongside individual care planning and individualized monitoring for glycemic control [42,43]. More frequent use of insulin pumps, continuous glucose monitoring, and smart meters with feedback systems was also found [43,47,48]. Positively reported to aid in diabetes management was the use and development of telemedicine, telehealth and telecare, TEC services, and Web platforms supported by the use of tools to communicate [30,45,47,49-52]. Two-way video links, virtual wards, and Android tablets allowed multidisciplinary team communication, integration, and care coordination [45-48] providing support for both patients and those caring for them. Environmental assistive devices, computers, trackers, monitors, and tablets are used to promote mental engagement with the outside communities and support networks including family [51], and they take a therapeutic approach with the aim to empower residents with a view to improve the overall quality of care and quality of life for residents in care homes [49,50].

### Limitations

Promoting systematic reviews as best practice has its challenges; it is thorough and therefore time-consuming as well as labor-intensive, requiring collaboration between authors. Data and methodology were poorly described in some studies and nonexistent in others; homogeneity from different study designs meant that we found considerable diversity of studies, reports, and case studies, which made it difficult to assess the risk of bias. Particularly, the limitation of the methodology used in this review was the information used for the analysis procedure based on the availability and quality of data to assess the risk of bias. However, some studies were written by experts in this field who were able to guide analysis and determine these objectives. Some included studies of qualitative nature were inappropriate for this approach, and therefore, the strengths of these studies needed to be balanced against the practical limitations of being able to determine the risk of bias; no studies were excluded on the basis of quality.

### Conclusions

To summarize on existing evidence and approaches found by conducting this review, there is limited published evidence of a standard practical role for technologies connecting the care home community with diabetes support, education, resources, and systems. There are many emerging forms of technology to enhance, support, and inform decisions about the management of residents’ diabetes care; however, there is no standardized approach to address access to specialist support or definitive standards of care in relation to diabetes management in the care home setting.

The use of technology has the potential to initiate early intervention, enable efficient responsive care, and most importantly, link the care home community to multidisciplinary clinical teams for support and communication. Therefore, standards need to be established with regard to care and management, with guidelines put in place. This includes mandatory educational frameworks for care home staff providing access to education and training so that the staff can maintain health and social care clinical competencies across the board. Improved diabetes education for care home staff is an important approach to improved diabetes management and the delivery of quality care.

Learning from examples of existing TEC and looking at new ways that health care technologies can help to provide a proactive approach in linking care homes with community health care services will assist in managing symptoms and reducing the impact of complications and hold potential to improve patient care pathways. Initiatives using technology to help support continuity of care for older people living with diabetes in the care home setting with complex care needs should be explored further.

Care homes play a vital role in the provision of support and care for most elderly members of our society for long-term care. Future aspirations for the care home setting are for care homes to be recognized as providers of safe, high-quality, individualized, and coordinated care. To achieve this fully, care home staff need to be supported in the delivery of excellent standards of care, being involved in new and creative approaches to the delivery of care. Nevertheless, engaging care home staff with a shared interest in improving the care of older people in care homes is multifaceted. Understanding how a new initiative or model of care will influence outcomes for care home residents has the potential to increase support for a change in practice.

## Appendix

Multimedia Appendix 1 Preferred Reporting Items for Systematic Review and Meta-Analysis (PRISMA P) 2015 checklist.

Multimedia Appendix 2 Data extraction form.

## Abbreviations

CGM: continuous glucose monitoring
e-learning: electronic learning
GP: general practitioner
GPS: Global Positioning System
HCP: health care professional
ICT: Information and Communication Technologies
MDT: multidisciplinary team
NHS: National Health Service
NP: nurse practitioner
PRISMA: Preferred Reporting Items for Systematic Review and Meta-Analysis
RCT: randomized controlled trial
RN: registered nurse
ROBIS: Risk of Bias in Systematic reviews
SBAR: Situation, Background, Assessment, Recommendation
TEC: technology-enabled care
VC: video conferencing
