# Supplementary material for: Diabetes, Care Homes, and the Influence of Technology on Practice and Care Delivery in Care Homes: Systematic Review and Qualitative Synthesis
Source: JMIR Diabetes. 2019 Apr 22;4(2):e11526. doi: 10.2196/11526 (PMC6658297; doi:10.2196/11526)
Supplement: Multimedia Appendix 2 [file diabetes_v4i2e11526_app2.pdf]

Appendix A. DATA EXTRACTION FORM

Author<br>Study year	
Country	
Paper No.	
Study design	
Aim/ discussion	
Analysis	
Setting	
Group covered/<br>Participants	
Type of technology<br>intervention	
Key findings, planned or<br>actual effectiveness<br>1	
2	
3	

4	
5	
Authors comments	Strengths<br><br>Weaknesses
Study Assessed Using ROBIS	
